# Annexin A2 and Kidney Diseases

**DOI:** 10.3389/fcell.2022.974381

**Published:** 2022-09-02

**Authors:** Ling Lin, Kebin Hu

**Affiliations:** Department of Medicine, The Pennsylvania State University College of Medicine, Hershey, PA, United States

**Keywords:** annexin A2, cell signaling, kidney diseases, renal inflammation, lupus nephritis, diabetic nephropathy (DN), renal cell carcinoma, nephrolithiasis

## Abstract

Annexin A2 is a Ca^2+^- and phospholipid-binding protein which is widely expressed in various types of cells and tissues. As a multifunctional molecule, annexin A2 is found to be involved in diverse cell functions and processes, such as cell exocytosis, endocytosis, migration and proliferation. As a receptor of plasminogen and tissue plasminogen activator, annexin A2 promotes plasmin generation and regulates the homeostasis of blood coagulation, fibrinolysis and matrix degradation. As an antigen expressed on cell membranes, annexin A2 initiates local inflammation and damage through binding to auto-antibodies. Annexin A2 also mediates multiple signaling pathways induced by various growth factors and oxidative stress. Aberrant expression of annexin A2 has been found in numerous kidney diseases. Annexin A2 has been shown to act as a co-receptor of integrin CD11b mediating NF-kB-dependent kidney inflammation, which is further amplified through annexin A2/NF-kB-triggered macrophage M2 to M1 phenotypic change. It also modulates podocyte cytoskeleton rearrangement through Cdc42 and Rac1/2/3 Rho pathway causing proteinuria. Thus, annexin A2 is implicated in the pathogenesis and progression of various kidney diseases. In this review, we focus on the current understanding of the role of annexin A2 in kidney diseases.

## Introduction

Annexins are a family of different calcium-regulated phospholipids-binding proteins, which were named for their ability to “annex” meaning aggregate membranes (Creutz et al., 1978; Bharadwaj et al., 2013; Luo and Hajjar, 2013). They are widely expressed in plants, fungi, invertebrates, vertebrates and others (Bharadwaj et al., 2013), and are found to be involved in the membrane related functions such as exocytosis, endocytosis, vesicular trafficking (Creutz et al., 1978; Bharadwaj et al., 2013; Luo and Hajjar, 2013). There are 12 annexin proteins in human, named as A1-A11 and A13, of which, annexin A2 is extensively studied. Human annexin A2 is a 36-kDa protein produced from *ANXA2* gene. It is expressed in endothelial cells, epithelial cells, monocytes, macrophages, dendritic cells and most cancer cells, and has been shown to be related to various human diseases (Rintala-Dempsey et al., 2008; Bharadwaj et al., 2013; Luo and Hajjar, 2013).

## Structure

Structurally, annexins typically have two main domains: a variable amino-terminal head domain and a homologous carboxyl core domain. The amino-terminal head domain contains sites for post-translation modification and is unique for individual annexins, allowing annexin to bind to various ligands or other proteins, and is responsible for distinct locations and specialized functions (Bharadwaj et al., 2013; Luo and Hajjar, 2013; Madureira and Waisman, 2013). The carboxyl core domain generally has four conserved structural repeat sequences, except annexin A6 which has eight repeat sequences. Each repeat sequence is formed from five α-helices, which play an important role in the structures of annexins through hydrophobic interactions between each other (Rintala-Dempsey et al., 2008; Bharadwaj et al., 2013; Luo and Hajjar, 2013).

Annexins bind to membranes reversibly in a calcium-dependent manner to perform their functions. Calcium ions bind to annexins on the same curves side of the annexin structures and form a convex surface which promotes the interaction of annexins and phospholipid. Though, after calcium binding, the annexins do not undergo a significant structural change within their core domains, they do have higher affinity to the anionic phospholipid-containing membrane (Swairjo et al., 1995; Rintala-Dempsey et al., 2008). In some annexins such as A1 and A2, the unique N-terminal sequences are also able to dissociate from the core domain on binding of calcium and will be closely associated with the core domain in the absence of the ion (Swairjo et al., 1995; Rintala-Dempsey et al., 2008).

Annexins, such as annexin A1 and A2, exist as free monomer or as heterotetramer through binding with S100A proteins. S100 proteins are dimeric EF-hand calcium-binding proteins. After coordinating calcium ions, they will undergo a significant conformational change to expose hydrophobic residues on their surface. These hydrophobic surfaces are able to bind two N-terminal sequences of annexin proteins and form a heterotetramer, which allows two membrane-bound annexin proteins to be brought into close proximity (Smith and Shaw, 1998; Santamaria-Kisiel et al., 2006; Rintala-Dempsey et al., 2008; Luo and Hajjar, 2013). Annexin A2 (ANXA2) was first identified as a substrate for the tyrosine kinase v-Src, a gene product found in Rous sarcoma virus, which mediates cellular transformation (Erikson and Erikson, 1980). Later, the structures and functions of the heterotetramer of ANXA2 and S100 proteins were extensively investigated. Since Rety and colleagues first revealed in 1999 that the first 13 residues of N-terminus of ANXA2 is responsible for its binding to S100A10 (p11) (Rety et al., 1999), ANXA2 has been found to bind to various S100 proteins including S100A4, S100A6, S100A11 and dicalcin, an S100-like protein (Filipek et al., 1995; Semov et al., 2005; Rintala-Dempsey et al., 2006; Uebi et al., 2007). Notably, S100A10 (p11), in the absence of calcium, maintains an EF-hand dimer conformation similar to the calcium-bound states of other S100 proteins (Bharadwaj et al., 2013).

## Signaling pathways and cellular functions

ANXA2 exists predominantly in cytoplasm and plasma membrane, and plays its functions through different mechanisms. ANXA2-S100A10 heterotetramer on the cell surface has been shown to be the receptor of plasminogen and tissue plasminogen activator (tPA); and plays an important role in the homeostasis of blood coagulation, fibrinolysis and matrix degradation, as well as mediating the cytokine effects of tPA *in vivo* (Dassah et al., 2009; Flood and Hajjar, 2011; Lin et al., 2012; Bharadwaj et al., 2013; Lin and Hu, 2017; Bharadwaj et al., 2021; Lim and Hajjar, 2021). ANXA2 is able to associate with anionic phospholipids, F-actin, and other proteins in the cell membrane and cytoskeleton, and regulates various membrane related functions including exocytosis, endocytosis, membrane bridging and tight junction formation (Yamada et al., 2005; Monastyrskaya et al., 2008; Su et al., 2010; Bharadwaj et al., 2013). The association is regulated by multiple factors including the calcium concentration, PH value and the post-translational modifications of ANXA2, implicating that ANXA2 mediates the effects of those factors in cell functions and processes. Due to the lack of intracellular domain, ANXA2, as a membrane-associate protein, can only dock onto the cell membrane in a peripheral manner (Kim and Hajjar, 2002). It remains largely unknown how ANXA2 transduces outside signals inside the cells. However, our recent work has demonstrated that ANXA2, upon binding of tPA, acts as a co-receptor of integrin CD11b, leading to their aggregation and activation of downstream integrin-linked kinase (ILK) and NF-kB signaling (Lin et al., 2012). Intriguingly, ANXA2 not only promotes NF-kB-dependent renal inflammation, but also mediates macrophage M2 to M1 phenotypic change leading to extensively enhanced inflammation (Lin and Hu, 2017). ANXA2, as an intracellular protein, modulates Rho signaling pathway through its interaction with cytoskeleton (Babbin et al., 2007; Ye et al., 2021), and regulates various membrane trafficking events (Luo and Hajjar, 2013), including calcium-mediated release of Weibel-Palade bodies (Knop et al., 2004), chromaffin granules (Umbrecht-Jenck et al., 2010), and lamellar bodies containing surfactant proteins (Wang et al., 2007). ANXA2 is also involved in the biosynthesis of cellular vesicles such as exosomes, and becomes a part of the cargos of these vesicles (Luo and Hajjar, 2013). Exosomes are excreted through an exocytotic process, and subsequently participate in intercellular interactions by contacting membrane of target cells and/or transferring their contents including ANXA2 (Margolis and Sadovsky, 2019). Thus, it is presumable that ANXA2 is involved in the extracellular vesicles (EV)-mediated cell-cell communication.

ANXA2 has several main phosphorylation sites including Tyr23, Ser25 and Ser11, which makes it a good candidate for the substrate of different kinases to participate in different signal pathways (Bharadwaj et al., 2013; Grindheim et al., 2017). Generally, Ser phosphorylation is involved in the regulation of S100A10 binding, secretory granules activity and mRNA binding, while Tyr phosphorylation is linked to actin dynamics and the endosomal pathway (Grindheim et al., 2017). Though ANXA2 is first found as a substrate of Src-family kinases, it also responds to the activation of numerous receptor tyrosine kinases by Tyr phosphorylation and involves in the signaling pathways of insulin, epithelial growth factor (EGF), platelet-derived growth factor (PDGF) and others (Brambilla et al., 1991; Biener et al., 1996; Rescher et al., 2008; Dziduszko and Ozbun, 2013; Cui et al., 2017). As a substrate of protein kinase C (PKC), ANXA2 contains sequence homology to the PKC binding site of 14-3-3 protein and may modulate PKC localization and signaling (Hoque et al., 2014). Moreover, increased Tyr 23 phosphorylation is also detected under the stress of heat, hypoxia, H_2_O_2_ and excitatory amino acids, suggesting that ANXA2 involves in the stress-induced signaling (Grindheim et al., 2017). In addition, ANXA2 contains four cysteine residues: Cys-8, Cys-132, Cys-261, and Cys-334, and is considered as a cellular redox regulatory protein and involved in redox-related functions on the cell surface, as well as in the cytoplasm and nucleus (Bharadwaj et al., 2013; Madureira and Waisman, 2013). For instance, Madureira and colleagues found that Cys-8 residue of ANXA2 is able to be oxidized by H_2_O_2_ and then reduced by the thioredoxin system, which results in the degrading of H_2_O_2_, a central redox signaling molecule mediating different signaling pathways upon its concentration *in vivo* (Madureira et al., 2011; Sies, 2017). On the other hand, oxidative stress-induced glutathionylation of Cys-8 and Cys-132 of the ANXA2 results in decreased binding of ANXA2-S100A10 heterotetramer with phospholipid and F-actin, and leads to related cellular function changes, suggesting that ANXA2 mediates oxidative stress-induced cellular function changes (Caplan et al., 2004).

## Annexin A2 and kidney diseases

Kidney disease is getting increasing recognition in the public health problems due to its increasing prevalence and high cost of treatment. It is also an important multiplier of risk of cardiovascular disease (Xu et al., 2018). It includes heterogeneous disorders with diverse etiologies affecting kidney structures and functions (Levey and Coresh, 2012). ANXA2, a calcium regulated phospholipids-binding protein, is expressed in various cells including macrophages, dendritic cells, endothelial and epithelial cells, and mediates various functions including endocytosis, exocytosis, cell-matrix interactions, cell motility, signal transduction, transcription, mRNA transport and DNA replication (Grindheim et al., 2017). Recent studies implicated that ANXA2 is an important participant in the pathogenesis of various kidney diseases.

### Annexin A2 and nephrotic syndrome

Components from a dysregulated immune system are able to mediate various acute renal injuries and play a central role in the progression of chronic kidney disease. Generally, auto-antibodies, produced through multiple mechanisms involving autoreactivity of both B and T cells, target various antigens within the kidney, lead damages directly at the local sites and finally result in the loss of kidney functions (Tecklenborg et al., 2018). ANXA2 is expressed in multiple cells in kidneys, therefore, auto-antibodies targeting ANXA2 may bind to the related cells and initiate directly renal damage through immune system.

Nephrotic syndrome is characterized by edema, proteinuria, hypoalbuminemia and hyperlipidemia, and is able to lead to end-stage kidney disease (Wang and Greenbaum, 2019; Politano et al., 2020). It may be primary or secondary to systemic disorders. The cause of primary nephrotic syndrome is not always clear, and immune dysregulation, systemic circulating factors, and structural and functional abnormalities of the podocyte can have deciding effects. Notably, some mutations in the genes encoding important podocyte proteins can cause defects in the glomerular filtration apparatus, resulting in nephrotic syndrome (Eddy and Symons, 2003; Noone et al., 2018).

Ye and colleagues has demonstrated that children with primary nephrotic syndrome (PNS) had higher level of anti-ANXA2 antibody, which significantly correlated with urine protein level. Intriguingly, BALB/c mice receiving intravenous injection of anti-ANXA2 antibody developed proteinuria as early as 24 h after injection. These mice displayed local foot-process fusion, basement membrane thickening, increased glomerular volume, narrowed balloon space, and large amount of collagen exudation. *In vitro* studies indicated that anti-ANXA2 antibody activates Cdc42 and Rac1/2/3 Rho pathway through phosphorylation of ANXA2 at Tyr24, leading to podocyte cytoskeleton rearrangement and eventually proteinuria (Ye et al., 2021).

### Annexin A2 and lupus nephritis

Systemic lupus erythematosus (SLE) is a multisystem autoimmune disease, which is characterized by a vast array of auto-antibodies, including anti-dsDNA antibodies. Auto-antibodies against nuclear and cellular antigens produced in SLE form immune complexes, which accumulate in the tissues and contribute to local damage (Seret et al., 2012). Lupus nephritis (LN), the most common cause of kidney injury of SLE, is a major risk factor for morbidity and mortality (Parikh et al., 2020). LN is characterized by the deposition of auto-antibodies in the mesangial area and along the glomerular basement membrane, complement activation, as well as the production of local mediators related to inflammation and fibrosis (Yung et al., 2010).

There are at least four different cell types in the glomeruli: mesangial cells, endothelial cells, podocytes and parietal cells, of which, mesangial cells, together with mesangial cell-specific antibodies, are thought to play central role in the pathogenesis of lupus nephritis (Seret et al., 2012). Among the numerous auto-antibodies, anti-dsDNA antibodies present in serum of nearly 80% of patients with LN and correlate closely with LN (Yung and Chan, 2015; Wang and Xia, 2019). Yung and colleagues isolated anti-dsDNA antibodies from 32 patients with significant IgG binding to human mesangial cells (HMC), and screened the proteins of the plasma membrane fraction of HMC that can bind to the antibodies directly. ANXA2 was identified among these proteins, and was found to mediate the binding of anti-dsDNA antibodies to HMC, which, in turn, induces IL-6 and ANXA2 expression in HMC. Prior incubation of anti-dsDNA antibodies with recombinant human ANXA2 inhibited the binding ability of the anti-dsDNA to HMC and the internalization of the anti-dsDNA by HMC. Glomerular ANXA2 expression correlated with LN severity (Yung et al., 2010). Similar screening in podocytes and glomerular proteins using the serum from patients with proliferative LN also identified ANXA2 as the antigen binding to the serum auto-antibodies (Caster et al., 2015). Their results are corroborated by several clinical studies in LN patients and experimental studies in mice, which demonstrated that serum level of ANXA2-binding immunoglobins, as well as expression of glomerular ANXA2, is associated with active LN progression (Ka et al., 2006; Salle et al., 2016; Zhou et al., 2018; Tesch et al., 2020). In a recent study regarding LN and T cell anti-renal autoreactivity, Tesch and colleagues identified ANXA2 as one of the main CD4+T cell targets in kidney (Tesch et al., 2020). Therefore, ANXA2, expressed in glomerular mesangial cells, is a pathogenetic target for the auto-antibodies in the SLE. ANXA2 mediates mesangial functions and local inflammation. ANXA2 is also one of the potential biomarkers for LN because its level correlates with the LN status (Ka et al., 2006; Salle et al., 2016; Cheung et al., 2017; Zhou et al., 2018).

### Annexin A2 and kidney inflammation

In the renal diseases initiated by the non-immunological mechanisms, such as acute kidney injury induced by artery infarction, the stress response pathways are activated and various cytokines and vasoactive factors are released upon the response of the renal epithelial cell damage, leading to the recruitment and activation of leukocytes and initiation of immune responses (Tecklenborg et al., 2018). Infiltrated leukocytes clear cellular debris and necrotic tissue, and promote renal healing process. However, in the presence of sustained severe damage or in the absence of anti-inflammatory factors, inflammatory process will be perpetuated and finally result in the tubular atrophy, interstitial scaring and worse kidney functions. Though the mechanisms of the regulation of the renal healing process is not completely clear, macrophages have been implicated (Rogers et al., 2014; Tecklenborg et al., 2018). Macrophage accumulation is one of the histological hallmarks of most interstitial and glomerular kidney diseases. In response to injury, macrophages differentiated into two broad but distinct categories as classically activated (M1) and alternatively activated (M2). Generally, M1 macrophages express a panoply of proinflammatory genes to promote inflammation and damage, however, M2 macrophages help to resolve inflammation and promote tissue remodeling (Ricardo et al., 2008; Wang and Harris, 2011; Lin and Hu, 2017).

Our studies have shown that tPA, a ligand of ANXA2, promotes kidney fibrosis and inflammation in the mice with unilateral ureteral obstruction (UUO), a classic chronic kidney disease mouse model. We found that tPA knockout mice had less collagen deposition, less CD11b positive macrophage infiltration, and less activation of NF-kB signaling, as indicated by lower renal p65 phosphorylation and less NF-kB-dependent chemokines, in the kidneys. *In vitro* studies discovered that ANXA2 mediates tPA-induced macrophage NF-kB activation through its aggregation and interaction with CD11b, followed by activation of its downstream mediator ILK (Lin et al., 2012). Further studies found that ANXA2-mediated NF-kB pathway promotes tPA-induced macrophage M2 to M1 phenotypic change, suggesting a pivotal role of ANXA2 in tPA-mediated renal inflammation (Lin and Hu, 2017).

Alternative pathway (AP) of complement is another contributor to kidney inflammation and is usually triggered by systemic illness or by tissue injury. Clinical and experimental studies implicate that AP activation contributes to the pathogenesis and promotes the injury of renal ischemia, focal segmental glomerulosclerosis (FSGS), antineutrophil cytoplasmic antibodies (ANCA)-associated vasculitis and lupus nephritis (Watanabe et al., 2000; Thurman et al., 2003; Thurman et al., 2005; Turnberg et al., 2006; Lenderink et al., 2007; Xiao et al., 2007; Xing et al., 2009; Noris et al., 2010). Factor H is a circulating protein which regulates the activation of AP of complement. Abnormal function of factor H is observed in numerous kidney diseases (Noris et al., 1999; Fremeaux-Bacchi et al., 2005; Servais et al., 2012; Sethi et al., 2012). To explore the mechanisms of factor H in the regulation of AP activation, Renner and colleagues screened the proteins from the post-ischemic kidney through immunoprecipitation with purified murine factor H, and identified ANXA2 as a factor H-binding partner. ANXA2 was shown to impair factor H function, increase complement activation on renal cell surface both *in vitro* and *in vivo*, and contribute to AP-mediated tissue inflammation (Renner et al., 2016).

### Annexin A2, nephrocalcinosis and nephrolithiasis

Nephrolithiasis and nephrocalcinosis are caused by the precipitation of poor soluble waste salts, such as calcium oxalate, in the renal tubular fluid. Calcium oxalate concentration is at low level in the ultrafiltrate, and increases gradually with oxalate secretion and water absorption, which results in the crystal formation in certain conditions. Crystal retention may result in kidney stones (nephrolithiasis) in the renal calyces and pelvis, as well as the tubular calcification (tubular nephrocalcinosis). Nephrolithiasis is associated with pain and suffering, and is responsible for about 10% of urological admissions per year. It affects 12% of men and 6% of women at some point in their lives. Tubular nephrocalcinosis can lead to obstruction-induced tubular injury and sometimes end-stage renal failure (Verkoelen and Verhulst, 2007).

Attachment of newly formed crystals to renal epithelial cells is critical to the development of kidney stone. Protein with high affinity to calcium oxalate monohydrate crystal was isolated from solubilized apical membrane proteins prepared from renal epithelial cells, and ANXA2 was identified using trypsin digestion and microsequencing technique. Further cellular experiments confirmed the expression of ANXA2 in the apical surface of renal epithelial cells. Pre-incubating with ANXA2-antibody blocked the binding of the crystals to renal epithelial cells. Therefore, ANXA2 may mediate adhesion of the crystals to tubular epithelial cells, promote crystal retention and finally contribute to the pathogenesis of nephrolithiasis and nephrocalcinosis (Kumar et al., 2003).

The role of ANXA2 in the crystal adhesion is also supported by other cellular and mouse studies. Dent’s disease is a genetic disorder caused by mutation of clc-5, a chloride channel, encoding gene and is characterized by low-molecular-weight proteinuria, hypercalciuria, nephrolithiasis and nephrocalcinosis. Disruption of clc-5 by transfection of antisense clc-5 or truncated clc-5 in a collecting duct cell resulted in the translocation of ANXA2 to plasma membrane through a mechanism to be further elucidated, which is accompanied with increased crystal binding and agglomeration. Pretreatment with anti-ANXA2 antibodies attenuated the effects of clc-5 disruption on crystal binding and agglomeration (Carr et al., 2006). In a mouse model of renal crystal formation induced by glyoxylate injection, it was found that fewer renal crystal deposits, accompanied with less ANXA2, were detected in oncostatin (OSM) receptor *ß* (OSMRβ)-deficient mice comparing to the wildtype controls (Yamashita et al., 2020).

### Annexin A2 and diabetic nephropathy

Diabetic nephropathy (DN) is the leading cause of end-stage renal disease (ESRD) worldwide. The pathogenesis of DN development and progression is complex with many contributing pathways and mediators, and remains not fully understood. It is known that persisting hyperglycemia-induced chronic glomerular hyperfiltration and excessive matrix deposition contribute to DN pathogenesis. However, current therapy with strict blood sugar and blood pressure control is unable to halt DN progression to ESRD (Samsu, 2021). KKAy mice, a crossbred between diabetic KK and lethal yellow (Ay), an obese gene, mice, develop obesity, dyslipidemia, insulin resistance and increased urine microalbumin levels spontaneously, and are widely used in the study of type 2 diabetes and DN (Okazaki et al., 2002). Proteomic profile in glomeruli of the KKAy mice with DN was analyzed by 2-dimensional differential gel electrophoresis and compared to C57BL/6 wide-type mice. ANXA2 and prohibitin, a protein interacting with ANXA2 upon the regulation of calcium, were identified and showed to be upregulated in the DN glomeruli, suggesting that ANXA2 may be associated with DN pathogenesis (Liu et al., 2014). *In vitro* study in human renal glomerular endothelial cells further validated that ANXA2 mediated mitomycin A-induced expression of collagen type VI and may contribute to DN pathogenesis since collagen type VI and phosphorylated histone H2AX, a DNA damage marker, were mainly detected in diabetic nodular glomerulosclerosis (Fujii et al., 2020). Dysregulation of the coagulation system and abnormal endothelial function exist in diabetic patients and are good predictors of DN (Alsharidah, 2022). ANXA2, as a receptor for plasminogen and tPA, plays an important role in maintaining vascular and hemostatic homeostasis (Lim and Hajjar, 2021). To explore the possible role of ANXA2 in DN pathogenesis and treatment, recombinant ANXA2 protein was administered into KKAy mice for 8 weeks with PBS administration as control. DN was alleviated after the ANXA2 protein treatment, as demonstrated by less kidney weight, lower level of albuminuria and smaller glomerular lesion area. However, there was no significant difference between the two groups in bleeding time, prothrombin time and active partial thromboplastin time, suggesting that ANXA2 protein alleviated DN progression with minimal influence on the coagulation system. Thus, ANXA2 administration may be a potential therapeutic tool for DN (Ishii et al., 2007).

### Annexin A2 and other kidney diseases

Acute tubular necrosis is the most common cause of the clinical state of acute renal failure. After injured by toxin, cells are damaged, cellular calcium is increased, quiescent cells undergo dedifferentiation, proliferation, and finally kidney function are restored (Toback, 1992). Calcium is an essential signaling molecular ion that controls a wide range of biological processes and functions, including cellular proliferation, differentiation and apoptosis. ANXA2, as an important calcium binding protein, was evaluated in uranyl nitrate-induced acute tubular necrosis. ANXA2 expression in renal cortex was increased in 3–7 days and attenuated on day 14. ANXA2 expression co-localized with proliferating cell nuclear antigen (PCNA). Similar ANXA2 expression pattern was observed in other acute tubular necrosis mouse models induced by folic acid and ischemic/reperfusion, though ANXA2 expression peaked at 12–24 h and declined in 72 h in the ischemic/reperfusion mouse model. It appeared that ANXA2 is the sensor of tubular injury and recovery in the acute renal failure (Cheng et al., 2005).

Mesangial proliferative glomerulonephritis is characterized by excessive mesangial cell proliferation and mesangial matrix expansion, which results in the glomeruli damage and loss of renal function. It may occur in IgA nephropathy, IgM nephropathy, lupus nephritis and other renal diseases. Proteomic analysis of renal tissue using iTRAQ technology uncovered that ANXA2 is upregulated in mesangial proliferative glomerulonephritis (Sui et al., 2013).

ANXA2 is also implicated in the cardiovascular diseases related to ESRD. Extensive and progressive vascular calcification is usually developed in ESRD patients. Lin and colleagues discovered that plasma exosomes derived from ESRD patients had higher level of ANXA2, which significantly promoted calcification of vascular smooth muscle cells (VSMCs), comparing to that from normal healthy group. Plasma exosome ANXA2 positively correlated to coronary artery calcification scores (Lin et al., 2020). Hyperphosphatemia, developed in chronic kidney disease and ESRD, is associated with increased cardiovascular risk. ANXA2 may also mediate the effect of phosphate in cardiovascular diseases, which was supported by the findings that ANXA2 was downregulated in human endothelial cells in exposure to high phosphate media or to serum from hyperphosphatemic patients, and that blockade of membrane-ANXA2 with a specific neutralizing antibody mimicked the effects of high phosphate, such as impaired endothelial cell migration and tube formation (Di Marco et al., 2013).

### Annexin A2 and renal cell carcinoma

Aberrant expression of ANXA2 has been found in numerous malignancies including gastric (Zhang et al., 2012), breast (Gibbs et al., 2020), lung (Yang et al., 2015a) and colorectal (Yang et al., 2013) cancer, as well as hepatocellular (El-Abd et al., 2016) and nasopharyngeal (Chen et al., 2018) carcinoma. ANXA2 has been implicated in cell cycle regulation and regulates tumor cell growth and survival. As a receptor for plasminogen and tPA, ANXA2 promotes plasmin generation, resulting in degradation of extracellular matrix (ECM), which facilitates cellular migration, tumor cell invasion and neoangiogenesis (Andreasen et al., 2000; Xu et al., 2015; Sharma, 2019). Moreover, ANXA2 Tyr 23 phosphorylation is implicated in the initiation of endothelial-mesenchymal transition (EndMT) and regulation of cell motility, and may contribute to tumor progression (Chen et al., 2018). ANXA2 also contributes to therapeutic resistance of multiple malignancies, such as nasopharyngeal carcinoma, gastric cancer, breast cancer and pancreatic cancer by mediating various signaling pathways including p38MAPK/Akt and PI3K/Akt/NF-kB pathways (Takano et al., 2008; Gong et al., 2010; Zhang et al., 2014a; Zhang et al., 2014b; Chen et al., 2015).

Renal cell carcinoma (RCC) is the ninth most common cancer worldwide and is the most common malignancy in adult kidney. Arising from nephric epithelial cells, RCC includes a group of heterogenous disorders characterized on the basis of anatomical origin, histological features, molecular hallmarks and therapeutic outcomes. The etiology is not fully understood. Multiple factors contribute to RCC pathogenesis, such as genetic predisposition, obesity, smoking, drugs and chemicals. Most of RCC patients are diagnosed incidentally during routine examination. Only up to 10% of RCC patients present typical clinical symptoms. Based on histological manifestation, RCC falls into three major subsets including clear cell RCC, papillary RCC, chromophobe RCC and several other rare subtypes such as collecting duct carcinoma, renal medullary carcinoma and urothelial carcinoma. Current treatment strategies for metastatic RCC includes cytoreductive nephrectomy, followed by immunotherapeutic drugs, antiangiogenic agents and mTOR inhibitors since RCC is resistant to traditional therapies (Yagoda et al., 1993; Tang et al., 2006; Jonasch et al., 2014; Petejova and Martinek, 2016; Wolf et al., 2020). Mechanistic studies of RCC pathogenesis, as well as searching for new diagnostic biomarkers and novel targeted therapies, were one of the hot spots over the past decades.

Tissue-associated genes were screened in the full-length enriched cDNA libraries of clear cell RCC and normal kidney tissues. Aberrant expression of ANXA2 in RCC was identified and validated by quantitative real-time PCR and Western blot (Tang et al., 2006). Since the tumor cells usually recapitulate embryonic cells, Sadashiv and colleagues investigated the ANXA2 expression in developing renal tissues and adult RCCs. Immunostaining revealed that ANXA2 was expressed in the ureteric buds and collecting tubules of fetal kidneys and in the collecting ducts of adult normal renal tissues, but not in the papillary RCC cells. Intriguingly, ANXA2 was also expressed in the proximal convoluted tubules, which is thought to be the origin of RCC, of younger fetal kidneys, and reappeared in clear cell RCC tumor cells, but not in that of normal adult kidneys. Proximal tubular expression of ANXA2 in fetal kidneys was more profound at earlier gestational weeks, and similar pattern was observed in the clear cell RCC with higher grade in tumor progression. Therefore, the deregulation of ANXA2 may be implicated in RCC pathogenesis (Sadashiv et al., 2019).

Since most RCC patients are detected incidentally during routine examination, earlier diagnosis remains challenging. Several studies investigated the relationship between ANXA2 expression and RCC development and progression, and explored the possibility of ANXA2 as a biomarker for RCC. Domoto and colleagues found upregulated expression of ANXA2 and its binding protein, S100A10, in RCC tissues. Compared to normal tissues, S100A10 and ANXA2 gene expression was 2.5-fold and 1.6-fold higher respectively in RCC. Immunostaining revealed that both S100A10 and ANXA2 were expressed in RCC tissues, but not in the proximal tubules of normal tissues, where most RCC derived from (Domoto et al., 2007). Similar results from Yang and colleagues further confirmed that ANXA2 expression was upregulated in all three major RCC subtypes (Yang et al., 2015b). More studies linked the expression of ANXA2 with RCC clinical manifestation and prognosis. Ohno and colleagues investigated the expression of ANXA2 in clear cell RCC tissues and normal tissues, and detected upregulated expression of ANXA2 gene and protein in 14 of 18 primary RCC tissues, positive ANXA2 immunostaining in 73 of 154 primary RCC tissues, as well as 21 in 24 metastatic tumors. In primary RCC, increased ANXA2 expression was significantly associated with higher stage and higher nuclear grade. The 5-years metastasis-free rate in patients with ANXA2-positive RCC was significantly lower than those with ANXA2-negative RCC, indicating that ANXA2 may be a predictor for metastasis and a useful marker for prognosis. It was also supported by a similar study in 33 RCC patients, in which ANXA2 expression was found to be correlated with Fuhrman grade and clinical outcomes (Zimmermann et al., 2004; Ohno et al., 2009).

Although multiple studies demonstrated that increased ANXA2 expression is associated with the poor prognosis of RCC, the underlying mechanisms remain unclear. Silencing ANXA2 in RCC cell lines resulted in decreased ability of cell migration and invasion, cell polarity alteration, disruption of actin filaments formation and reduced CXCR4 expression. Inhibition of Rho/Rock axis restored cell motility suppressed by ANXA2 silencing suggesting that Rho/Rock is involved in ANXA2 signaling pathways (Yang et al., 2015b). Soluble ANXA2 was detected at higher level in renal vein serum from some RCC patients and was proved to inhibit phytohaemagglutinin-induced lymphoproliferation *in vitro,* suggesting immunosuppression effect of ANXA2 in the RCC progression (Aarli et al., 1997). Increased ANXA2 was detected in the rat RCC induced by ferric nitrilotriacetate, an iron chelate inducing renal oxidative damage. Further investigation showed that ANXA2 was increased time-dependently in the rat kidney after ferric nitrilotriacetate administration, as well as in H_2_O_2_-treated LLC-PK1 cells, a normal renal tubular cell line. The pattern of ANXA2 expression in rat was similar to that in clinical patients, as higher expression of ANXA2 was detected in regenerating proximal tubules, primary and metastasizing RCCs, comparing to normal kidney. Serine and tyrosine phosphorylation was also detected. Overexpression of ANXA2 induced LLC-PK1 cell proliferation, while silencing ANXA2 decreased the proliferation of FRCC cells derived from ferric nitrilotriacetate-induced primary RCCs (Tanaka et al., 2004). Therefore, it is presumable that ANXA2 mediates oxidative stress-induced renal carcinogenesis and metastasis.

## Discussion

ANXA2, as a multifunctional molecule, regulates diverse cellular functions and processes through various signaling pathways. ANXA2 antibody, gene silencing techniques and ANXA2 protein administration have been used in cellular and animal experiments to explore the role of ANXA2 in kidney diseases, as well as the underlying mechanisms. As shown in Table 1, aberrant expression of ANXA2 has been detected in numerous kidney diseases, including primary and secondary nephropathy, acute kidney injury, chronic kidney disease, as well as kidney carcinoma. ANXA2, expressed in the mesangial cells and podocytes, acts as the antigen for the auto-antibodies in patients, mediates renal inflammation and injury in LN and PNS. ANXA2 also promotes kidney inflammation through mediating tPA-induced NF-kB activation and M2 to M1 phenotypic change, as well as binding to factor H to impair function of factor H and increase AP activation. ANXA2 mediates the binding of calcium oxalate crystals to renal epithelial cells and is involved in the pathogenesis of nephrolithiasis and nephrocalcinosis. ANXA2 participates in the pathogenesis and progression of RCC through its effects on immunosuppression, cell proliferation, migration and invasion. Studies revealed that ANXA2 expression correlates with the disease status of LN and RCC, implicating that ANXA2 may be a potential biomarker for RCC and LN. The fact that ANXA2 protein administration alleviated DN in the KKAy mice model suggests that it may be a potential therapeutic tool for DN. Of note, the current knowledge regarding the role of ANXA2 in kidney diseases is very limited, and many of the publications are association studies. Therefore, future more definitive and mechanistic studies on the role of ANXA2 in the pathogenesis of kidney diseases are warranted.

**TABLE 1:** Summary of the roles of annexin A2 in kidney diseases. Annexin A2 executes multiple cellular process and signaling pathways to participate in the pathogenesis and progression of various kidney diseases.

Diseases	Effects/Changes of ANXA2	References
Primary Nephrotic Syndrome	Podocyte anti-ANXA2-mediated Rho signaling	Ye et al. (2021)
Lupus Nephritis	Auto-antibodies binding to glomerular ANXA2 and up-regulation of ANXA2	Ka et al. (2006); Yung et al. (2010); Caster et al. (2015); Yung and Chan (2015); Salle et al. (2016); Zhou et al. (2018)
	ANXA2 as target of CD + T cell	Tesch et al. (2020)
Kidney Inflammation	Mediates tPA-induced NF-kB activation and promotes macrophage M2 to M1 phenotype	Lin et al. (2012); Lin and Hu (2017)
	Impairs factor H function	Renner et al. (2016)
Nephrocalcinosis and Nephrolithiasis	Mediates adhesion of the crystals to tubular epithelial cells	Kumar et al. (2003); Carr et al. (2006); Yamashita et al. (2020)
Diabetic Nephropathy	Increased glomerular ANXA2 expression in DN	Liu et al. (2014)
	Recombinant ANXA2 alleviates DN	Ishii et al. (2007)
Acute Tubular Necrosis	Increased ANXA2 expression in renal cortex	Cheng et al. (2005)
Mesangial Proliferative Glomerulonephritis	Increased mesangial ANXA2 expression	Sui et al. (2013)
ESRD Associated Cardiovascular Diseases	Pro-calcification of vascular smooth muscle cells	Lin et al. (2020)
	Mediates the effects of phosphate	Di Marco et al. (2013)
Renal Cell Carcinoma (RCC)	Increased ANXA2 expression in RCC	Zimmermann et al. (2004); Tang et al. (2006); Domoto et al. (2007); Ohno et al. (2009); Sadashiv et al. (2019)
	Promotes migration, invasion and proliferation	Tanaka et al. (2004); Yang et al. (2015b)
	Immunosuppression effect	Aarli et al. (1997)
